# Revised one-bag IV fluid protocol for pediatric DKA: a feasible approach and retrospective comparative study

**DOI:** 10.1093/tropej/fmae003

**Published:** 2024-02-09

**Authors:** Durmuş Doğan, Hatice D C Gökalp, Erdal Eren, Halil Sağlam, Ömer Tarım

**Affiliations:** Department of Pediatric Endocrinology, School of Medicine, Çanakkale Onsekiz Mart University, Çanakkale, Türkiye; Department of Pediatric Medicine, Pediatric Endocrinology, Bursa City Hospital, Bursa, Türkiye; Department of Pediatric Endocrinology, School of Medicine, Bursa Uludag University, Bursa, Türkiye; Department of Pediatric Endocrinology, School of Medicine, Bursa Uludag University, Bursa, Türkiye; Department of Pediatric Endocrinology, School of Medicine, Bursa Uludag University, Bursa, Türkiye

**Keywords:** dextrose, diabetic ketoacidosis, one-bag protocol, pediatrics

## Abstract

**Background:**

This study compared the effectiveness of the traditional and revised one-bag protocols for pediatric diabetic ketoacidosis (DKA) management.

**Methods:**

This single-center retrospective cohort study included children diagnosed with DKA upon admission between 2012 and 2019. Our institution reevaluated and streamlined the traditional one-bag protocol (revised one-bag protocol). The revised one-bag protocol rehydrated all pediatric DKA patients with dextrose (5 g/100 ml) containing 0.45% NaCl at a rate of 3500 ml/m^2^ per 24 h after the first 1 h bolus of normal saline, regardless of age or degree of dehydration. This study examined acidosis recovery times and the frequency of healthcare provider interventions to maintain stable blood glucose levels.

**Results:**

The revised one-bag protocol demonstrated a significantly shorter time to acidosis recovery than the traditional protocol (12.67 and 18.20 h, respectively; *p* < 0.001). The revised protocol group required fewer interventions for blood glucose control, with an average of 0.25 dextrose concentration change orders per patient, compared to 1.42 in the traditional protocol group (*p* < 0.001). Insulin rate adjustments were fewer in the revised protocol group, averaging 0.52 changes per patient, vs. 2.32 changes in the traditional protocol group (*p* < 0.001).

**Conclusion:**

The revised one-bag protocol for pediatric DKA is both practical and effective. This modified DKA management achieved acidosis recovery more quickly and reduced blood glucose fluctuations compared with the traditional one-bag protocol. Future studies, including randomized controlled trials, should assess the safety and effectiveness of the revised protocol in a broad range of pediatric patients with DKA.

## INTRODUCTION

Treatment of diabetic ketoacidosis (DKA) involves administering intravenous fluids (IVF), insulin and electrolytes to correct metabolic abnormalities and restore normal acid-base, electrolyte and glucose balances [1]. Excess fluid administration and osmotic fluid shifts cause cerebral edema, influencing DKA protocols [2]. However, protocols developed using this knowledge are complex and difficult to interpret [3, 4].

The traditional approach to fluid therapy for DKA is a one-bag protocol, which involves supplying IVF from a single bag and insulin from another bag [5]. Initially, the IVF contains no dextrose (only electrolyte) and is administered until serum glucose declines to 250–300 mg/dl, after which dextrose is added. However, this approach requires frequent insulin and fluid therapy adjustments to accommodate fluctuations in patients’ electrolyte, fluid and dextrose requirements. Multiple IVF bag changes during DKA management limit variations in fluids administered fluids and increase costs. The two-bag protocol, which involves two bags with identical electrolyte contents but different dextrose concentrations (0% and 10%), is recommended for more precise and flexible glucose concentration adjustments in IVF therapy [6]. However, the one-bag method is still commonly used in Turkey.

The traditional one-bag protocol was employed at our university until 2015. Physicians experienced difficulty calculating fluid volume, changing IVF bags and maintaining potassium levels within the normal range. Therefore, we revised our one-bag protocol to simplify fluid calculations and reduce IVF bag changes to control fluctuations in dextrose requirements. Previous reports have indicated that rehydration exceeding 4000 ml/m^2^ per 24 h may pose a risk for the development of cerebral edema [7]. Therefore, various fluid regimens have been proposed to mitigate this risk [8, 9]. We assessed a daily rehydration volume of 3500 ml/m^2^ for safe fluid administration, for each patient, considering maintenance and deficit. Simulations using both protocols confirmed the adequacy of the volume, eliminating the risk of exceeding 4000 ml/m^2^. Our revised protocol involves administering 0.45% saline with 5% dextrose (75 mEq/l NaCl, 5 g/100 ml dextrose) (D5W NS) at 3500 ml/m^2^ every 24 h after the initial 1 h bolus of normal saline (NS), regardless of the degree of dehydration, age, or glucose levels in all DKA cases. This simplifies the fluid volume calculation and decision-making process. Given the frequency of hypokalemia, we used 50 mEq/l potassium in revised one-bag protocol.

We developed this protocol based on our clinical observations and the need for a more practical and adaptable approach for pediatric DKA management. We believe it is a valuable contribution to the field and our study provides comprehensive details on its implementation and outcomes.

The primary outcome was acidosis recovery time (resolution of acidosis), whereas the secondary outcomes were insulin rate, fluid dextrose content changes and incidence of complications.

This study aimed to compare the effectiveness of traditional and revised one-bag protocols for pediatric DKA management, examining acidosis recovery times and the frequency of healthcare provider interventions to maintain stable blood glucose levels during treatment.

## METHODS

This study was conducted at a tertiary care referral hospital in Turkey's Southern Marmara region and approved by the Clinical Research Ethics Committee (2015-14/13 and 2023/03-07). Patients aged <18 years with an admission diagnosis of DKA were retrospectively identified from medical records from February 2012 to January 2016 for the traditional one-bag protocol and from November 2016 to February 2019 for the revised one-bag protocol. The transitional year (February–November 2016) was excluded. The diagnosis of DKA was verified based on blood glucose >200 mg/dl, pH<7.30, bicarbonate <15 mmol/l and the presence of moderate or severe ketonuria [1]. DKA severity was classified based on the degree of acidosis: Mild DKA; venous pH <7.3 and/or HCO_3_ < 15 mmol/l, moderate DKA; venous pH <7.2 and/or HCO_3_ <10 mmol/l and severe DKA; venous pH <7.1 with or without HCO_3_ < 5 mmol/l [10]. Patients with mild DKA treated with subcutaneous insulin, whose treatment started outside our hospital, and whose medical records had insufficient data were excluded. DKA episodes treated during the transitional year were excluded.

Data were extracted from the medical records at admission, including age, sex and biochemistry (glucose, sodium, potassium, urea, creatinine, pH, bicarbonate and partial carbon dioxide pressure). Acidosis recovery time (time to resolution of acidosis) was determined as the duration from the initiation of the NS bolus to pH >7.3 and bicarbonate >15 mEq/l, in blood gas analysis [11].

### Interventions

Patients were evaluated in the emergency room, and patient who required an insulin drip were automatically transferred to the intensive care unit. Upon presentation to the emergency department, all patients diagnosed with DKA received a 10 ml/kg bolus infusion of NS over 60 min.

### Traditional one-bag protocol

In the traditional one-bag protocol, the physician calculates fluids based on clinically confirmed dehydration (7–10%, on initial presentation) [12]. The Holliday–Segar method was used to calculate the daily maintenance fluid, and the deficit with the 48 h maintenance fluid determined the rehydration fluid volume. NS was the preferred rehydration fluid and was administered at a constant infusion rate for 48 h. When blood sugar dropped below 300 mg/dl, the rehydration fluid was changed to D5W NS. Insulin was administered concurrently with the rehydration fluid at a rate of 0.1 units/kg/h after first 1 h of bolus NS infusion.

We initially increased fluid dextrose concentrations to prevent hypoglycemia (or glucose declined by >75 mg/dl/h), progressing from 7.5% to 10% or 12.5%, then insulin was reduced by 10%. If blood glucose increased or did not decrease, we increased the insulin dose by 10% and continued to adjust according to the response. Potassium replacement was initiated at 40 mEq/l (Table 1). Target potassium was 3.5–5.5 mEq/l. The rehydration fluid contained 40–60 mEq/l potassium to maintain this range.

**Table 1.** Feature of the traditional and revised one-bag protocols

	Traditional protocol	Revised protocol
Initial saline bolus	10 ml/kg, 1 h	10 ml/kg, 1 h
Rehydration fluid volume	[2 × maintenance (rehydration) + deficit]/48 h	3500 ml/m^2^ per 24 h
Rehydration fluid administration	NS until blood sugar <300 mg/dl Add dextrose (D5W NS^a^) Check volume, 4000 ml/m^2^ per 24 h	D5W NS^a^
Insulin	0.1 units/kg/h	0.1 units/kg/h
Potassium	40 mEq/l	50 mEq/l

a D5W NS, 5% dextrose + 0.45% NaCl; NS, normal saline.

### Revised one-bag protocol

In the revised one-bag protocol, the rehydration protocol involved administering a constant infusion rate of dextrose-containing fluid after the initial NS bolus. The rehydration fluid was a ready-to-use fluid preparation containing D5W NS, administered at a rate of 3500 ml/m^2^ per 24 h for all DKA episodes. The infusion rate and dextrose concentration remained uniform and were not adjusted for the degree of dehydration, age or initial glucose level. Insulin therapy was administered concurrently with rehydration fluid at a rate of 0.1 units/kg/h. In this group, potassium replacement was initiated with 50 mEq/l potassium chloride (Table 1). The interventions for insulin and dextrose concentration titrations and achieving the target potassium were similar to those in the traditional one-bag protocol group.

### Monitoring

Clinical and biochemical monitoring was performed in accordance with the International Society for Pediatric and Adolescent Diabetes clinical practice consensus guidelines [1]. As clinically indicated, serum electrolytes, glucose and blood gases were repeated every 2–4 h or more frequently. Blood ketone concentrations were not used for monitoring. Hourly vital signs, capillary blood glucose and neurological observations were recorded. Our clinic identifies symptoms like headache, vomiting, altered consciousness, changes in Glasgow Coma Scale score and new incontinence, bradycardia or hypertension post-treatment initiation as suspicious cerebral edema. Hypertonic saline was used for suspected cases, with brain imaging performed for non-responsive cases.

### Types and numbers of changes in treatment

A smaller cohort was examined to assess the physicians’ efforts to adjust electrolyte, fluid and dextrose requirements during treatment. A detailed review of patient files from a single year of each protocol’s implementation was performed (sub-cohort analysis). This approach aimed to minimize data loss and errors in the retrospective analysis. Patient files and treatment charts were independently reviewed by two physicians and every intervention was meticulously recorded. For instance, if a physician made three changes to the insulin infusion rate for a case, it was recorded as three separate changes. This level of detail allowed accurate capture of the dynamics of clinical decision making. Both protocols required adjustment of the IV fluid bags to achieve precise dextrose concentrations ranging from 0% to 12.5% obtained from a single bag. Consequently, each dextrose concentration modification resulted in an increased number of IV fluid bags. Discrepancies or ambiguities in the intervention records were resolved by consensus between the two reviewing physicians, ensuring the robustness of our data.

### Outcomes

This study rigorously assessed two crucial outcomes to gauge the effectiveness and safety of the revised one-bag protocol compared with the traditional one-bag protocol. The primary outcome was acidosis recovery time, with comparisons made within both protocol groups and DKA severity subgroups. Additionally, secondary outcomes were assessed, including number of changes to insulin rate, number of changes to fluid dextrose orders and incidence of complications.

### Sample size

To determine the sample size, we formed a small group of 10 patients from each protocol group. Acidosis recovery times were documented; for the classic protocol a mean of 21.39 h with an SD of 12.5, and for the revised protocol a mean of 17.4 h with an SD of 9.25. A G-power analysis yielded an effect size of ∼0.35. Subsequently, we applied this effect size, a significance level of 0.05, and a power of 0.80 to calculate that a minimum of 110 records would be necessary for each protocol group. This suggests that 2–3 protocol years per group was sufficient.

### Statistical method

Data are reported as median (25th–75th quartile) or mean ± SD. Normality for both primary and secondary outcomes was assessed using the Shapiro–Wilk test. Based on normality, continuous variables were compared between groups using either the Mann–Whitney U-test or *t*-tests. Categorical variables were analyzed with the chi-square test, or Fisher’s exact test if cell frequency was <5. Statistical analyses used IBM SPSS Statistics (version 23.0, Armonk, NY, USA), with *p* < 0.05 as the significance threshold.

## RESULTS

**Figure 1:**
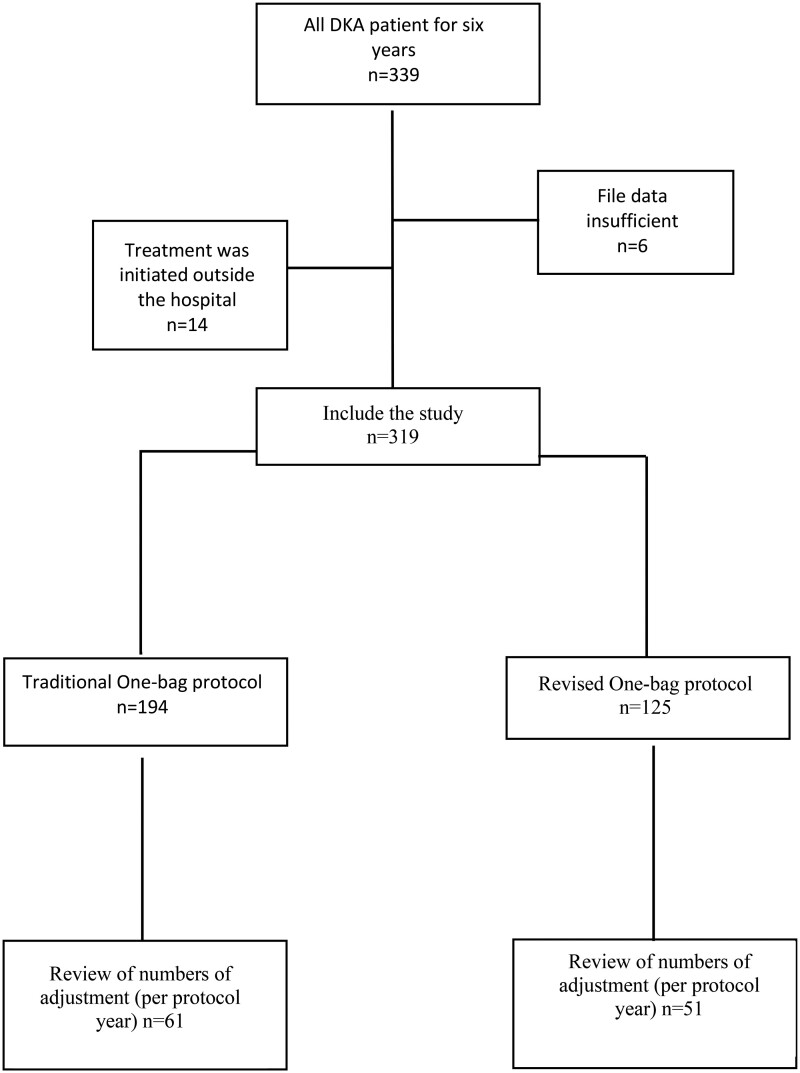
Flow chart of the retrospective cohort study to compare traditional one-bag and revised one-bag DKA protocols.

A total of 339 files were screened for this study. After exclusions, 319 files of DKA met the inclusion criteria. Of these, 194 were treated with the traditional one-bag protocol and 125 were treated with the revised one-bag protocol (Figure 1). No patients required intubation and no neurological sequelae were reported in either treatment group.

**Figure 2:**
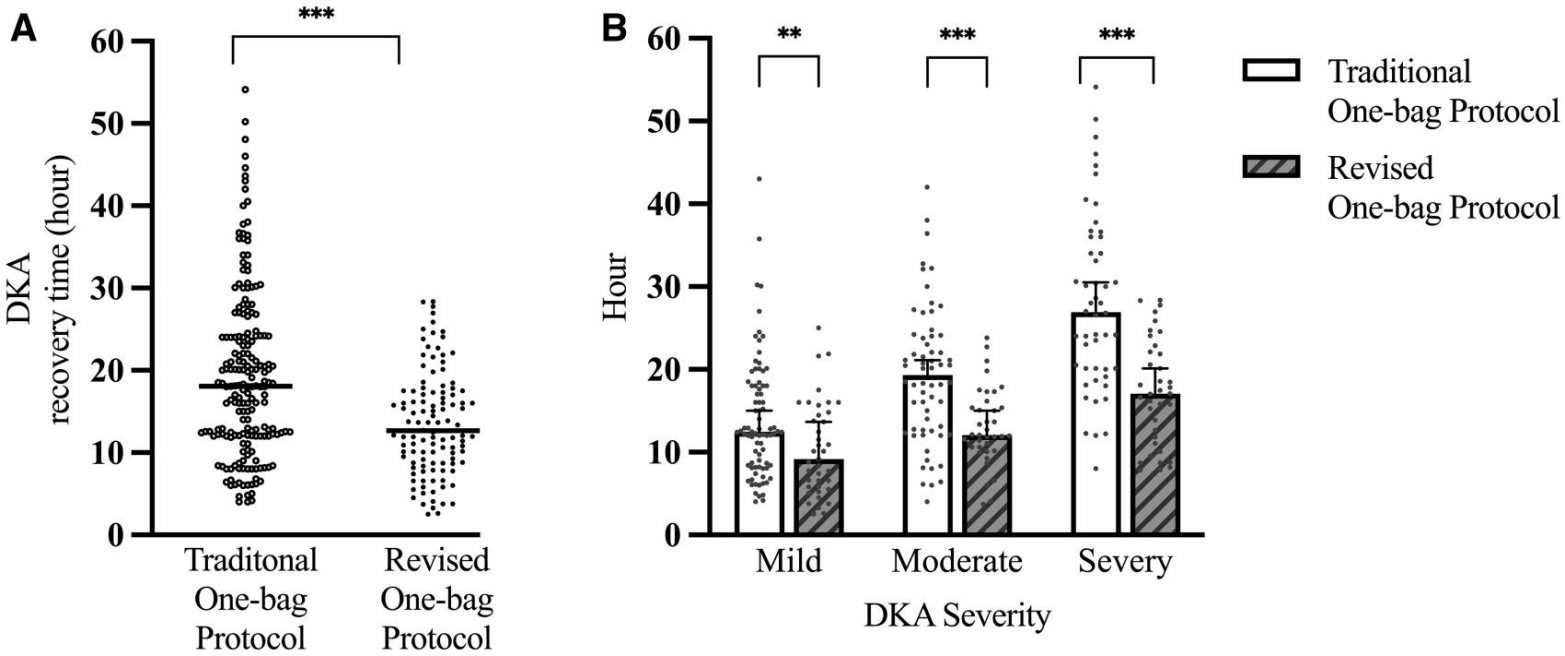
Acidosis recovery time in traditional and revised one-bag groups. (**A**) All patients in each protocol group. (**B**) Subgroups of DKA severity in each protocol group (**p < 0.001, ***p < 0.0001).

Table 2 shows the baseline characteristics of the patients were similar, except for potassium and creatinine. Median (25th–75th) recovery time of acidosis was 18.02 h (11.8–24.1) in the traditional protocol group and 12.67 h (8.76–17.33) in the revised protocol group (*p* < 0.001). Mild, moderate and severe DKA episodes in which the revised protocol was applied improved significantly (Figure 2).

**Table 2.** Characteristics of the groups; age, gender and initial laboratory findings

	**Traditional protocol** **(*n*=194)**	**Revised protocol** **(*n* = 125)**	*p*
Age (years)	11.08 (7.56–13.35)	11.66 (7.83–13.91)	0.134^a^
Gender (M, F)	84, 110	55, 58	0.362^b^
Glucose (mg/dl)	462 (372–558)	478 (391–567)	0.445^a^
pH	7.17 (7.08–7.17)	7.16 (7.07–7.22)	0.261^a^
Bicarbonate (mmol/l)	8.65 (6.2–12.2)	8.20 (5.35–11.3)	0.150^a^
pCO_2_	23.60 ± 6.18	22.78 ± 7.37	0.316^c^
Na^+^ (mmol/l)	132 (128–134)	131 (128–134)	0.100^a^
K^+^ (mmol/l)	4.40 (3.9–4.8)	4.56 (4.1–5.1)	0.018^a^
Urea (mg/dl)	29 (21–38)	27 (18–36)	0.266^a^
Creatinine (mg/dl)	0.90 (0.8–1.1)	0.98 (0.84–1.12)	0.008^a^
Severity DKA (*n*, %)			
Mild DKA	82 (42.3)	39 (34.5)	
Moderate DKA	62 (32)	37 (32.7)	0.312^b^
Severe DKA	50 (25.8)	37 (32.7)	

Data were presented as median (25th–75th) or mean ± SD.

a Mann–Whitney U-test.

b Chi-square test.

c Independent samples *t*-test.

Assessing the physician’s efforts, we analyzed files from the 1 year periods that followed and preceded the transition year (Figure 1). In the sub-cohort analysis, only bicarbonate was significantly lower in the revised protocol in 2016 compared to the traditional protocol in 2014 [7.35 mmol/l (4.85–10.95) vs. 9.70 mmol/l (7.15–11.50), respectively; *p* = 0.027] (Supplementary Material). All other initial parameters, including age, sex, glucose, pH and electrolytes, showed no statistically significant differences.

The most common change in both groups was increased dextrose concentration. Changes in IV fluid dextrose concentration were required in 60 (98.4%) of the 61 patients in the traditional protocol group and only 10 (19.6%) of the 51 patients in the revised protocol group. In addition, the number of fluid dextrose changes per patient was 1.42 in the traditional protocol group and 0.25 in the revised protocol group (*p* < 0.001). Most changes were switching from 0.9% NaCl to D5W NS when blood glucose was <300 mg/dl in the traditional protocol group. Even when this last situation was not considered, the number of fluid orders in the revised protocol group was 42% lower than in the traditional protocol group (*p* = 0.036). The number of changes in insulin rate per patient was 2.32 in the traditional protocol group and 0.52 in the revised protocol group (*p* < 0.001) (Table 3).

**Table 3.** Comparison of the number of changes in orders of the dextrose concentration and insulin infusion rate in traditional and revised protocol groups

	**Traditional protocol** **(*n*=61)**	**Revised protocol** **(*n*=51)**	*p*
Number of patients needing dextrose adjustment (*n*, %)	60 (98.4%)	10 (19.6%)	<0.001^a^
Number of changes to IV dextrose orders per patient	1.46	0.21	<0.001
IV dextrose change orders per patient (excluding transition from NS)^b^	0.37	0.21	0.036
Number of patients needing insulin adjustment (*n*, %)	39 (63.9%)	13 (25.5%)	<0.001^a^
Number of changes to insulin rate per patient	2.32	0.52	<0.001

a Chi-square test.

b Excluding IV fluid changes during the transition from isotonic saline to dextrose in the traditional protocol.

In the revised protocol group, where the initial potassium replacement was 50 mEq/l, hypokalemia was observed in a smaller proportion (*n* = 13, 25%) compared to the traditional one-bag protocol group, where the initial potassium replacement was 40 mEq/l (*n* = 40, 65%) (χ^2^=17.9, df = 1, *p* < 0.001). Hyperkalemia was observed in two patients in the revised protocol group, with one instance detected at initial potassium, and one case in the traditional one-bag protocol group.

Brain edema, confirmed by imaging, was absent in both groups. Hypertonic saline was administered to two patients (1%) in the traditional group and to three in the revised group, with no neurological deficits or mortality observed in any of the patients.

## DISCUSSION

This study compared the clinical outcomes of two pediatric DKA protocols in a single tertiary care center. We showed that the revised one-bag protocol resolved acidosis faster than the traditional protocol. Physicians required fewer fluid dextrose and insulin adjustments during DKA treatment with the revised protocol.

In current DKA protocols, variations in interpretation among physicians often arise due to subjectivity and inaccuracy of calculating dehydration degrees and volume deficits [13, 14]. This complexity can lead to clinical errors and increase risk of brain edema, emphasizing the importance of avoiding hypotonic fluids, excessive fluid administration and rapid glucose reduction. To address these concerns, we adapted the traditional protocol to a revised one-bag protocol. The fluid will never exceed 4000 ml/m^2^ per 24 h, ensuring safe hydration in all cases and avoiding complex calculations. The revised protocol formulates fluids 10% safer than the traditional one-bag protocol (Table 4). Fluid administration in the revised protocol may have faster renal excretion of accumulated organic acids compared with the traditional one-bag protocol. This may explain the faster resolution of acidosis. Rewers, *et al.* [15] conducted a large randomized clinical experiment to compare acidosis correction and electrolyte normalization rates with different fluid infusion rates and NaCl concentrations. They showed faster fluid administration normalized the anion gap 2–3 h before slower fluid infusion rates.

**Table 4.** Comparison of the type of fluid, volumes and infusion rates to be given in the case of traditional and revised protocols applied to a sample patient weighing 30 kg (body surface area of 1 m^2^)

	**Traditional protocol** ^a^		Revised protocol	
Time	Fluid type	ml/h	Fluid type	ml/h
0–1 h	NS	300	NS	300
1–4 h	NS	133	D5W NS	145
4–24 h	D5W NS	133	D5W NS	145
24 h total fluid (ml)	3450		3800	

a Calculated as 10% dehydrated. D5W NS, 5% dextrose + 0.45% NaCl; NS, normal saline.

The Dallas protocol is remarkable because of its simplicity and applicability to the treatment of DKA. All patients receive 2.5 times the maintenance rehydration therapy, regardless of the degree of dehydration, and a three-bag procedure containing two bags of rehydration fluid and one bag of insulin [8]. The protocol allowed for 30% more fluid and 70% more sodium, reducing DKA resolution time from 16 to 12 h. Another study compared the Dallas protocol with the recommendations of the American Diabetes Association’s (ADA-2006) recommendations and observed that it resulted in lower mortality and fewer disabilities [16]. This protocol prevents calculation errors and improves blood sugar control, resulting in shorter acidosis duration and lower therapeutic costs [8].

The revised one-bag protocol effectively reduces fluid and electrolyte adjustments, simplifying pediatric DKA treatment. Using a fluid bag containing dextrose after the initial NS bolus without waiting for blood glucose to reach 300 mg/dl is important. When blood glucose drops below 300 mg/dl in the traditional single-bag protocol group, NS was replaced with D5W NS. Even without this criterion, the revised one-bag protocol significantly reduces dextrose adjustment order compared with the traditional one-bag protocol group. Moreover, the revised one-bag protocol significantly reduced insulin rate changes per patient. Reducing dextrose change and insulin adjustments may have contributed to the stabilizing of glucose fluctuations in the revised protocol.

Managing DKA presents a challenge to achieve the desired potassium levels. Although administration of 40 mEq/l potassium is recommended, the effects of this concentration are largely unknown. Our study reported that patients starting with 40 mEq/l potassium showed more hypokalemia, and less at 50 mEq/l. However, there are insufficient studies on higher doses in the literature. Basnet, *et al.* [17] investigated the effect of potassium infusions on serum potassium in children with DKA using 40 and 60 mEq/l based on initial serum potassium. They reported that hypokalemia was more common in the low-potassium group receiving 60 mEq/l. The authors suggested that a 60 mEq/l infusion may benefit children with DKA presenting with a lower baseline serum potassium, although more research is needed for validation [17]. Further studies are needed to determine how potassium infusion doses affect serum potassium in children with DKA.

### Limitations and future studies

Our study has certain limitations. First, this was a single-center, retrospective study. Therefore, our findings may not apply to larger populations. Selection bias and variable uncontrollability were created by a retrospective design. Second, owing to the retrospective nature, despite careful review of patient files and orders, certain interventions or details may have been missed or recorded inaccurately.

Third, our study focused on the primary outcome, time to recovery from acidosis and secondary outcomes, including alterations in insulin rates and dextrose content. This limited scope may have prevented identification of all DKA management variables, including hyperchloremic metabolic acidosis and other electrolyte abnormalities. Additional complexity arose because of the use of two different potassium levels in the study, 40 and 50 mEq/l. This variation complicates a direct comparison between the two groups, potentially affecting the conclusions of the study on electrolyte management in DKA.

Fourth, our study compared one-bag DKA treatment protocols and not with two-bag protocols. We did not analyze cost-effectiveness, although the revised one-bag protocol’s reduced insulin rate and dextrose concentration modifications may suggest cost-efficiency.

Fifth, our cohort predominantly consisted of patients with mild-to-moderate DKA (65–70%). Therefore, our findings may be most applicable to this patient subset. However, this protocol may have limited applicability in cases of severe DKA and requires further validation.

Lastly, we acknowledge that blood ketone levels and hyperchloremia can provide additional insights into the management of ketoacidosis, and it is an aspect that could be measured and assessed in future studies.

Future research should assess cost-effectiveness and hospitalization duration. Randomized controlled trials (RCTs) comparing the revised one-bag protocol, the traditional one-bag protocol and the two-bag protocol can provide stronger conclusions regarding safety and effectiveness. RCTs would explore how patient-specific factors, such as age, DKA severity and initial metabolic levels, affect treatment outcomes. Future studies should examine risks, such as hyperchloremia, hypoglycemia and acute kidney problems, during treatment.

## CONCLUSION

We demonstrated that the revised one-bag protocol resolves acidosis faster and requires fewer fluid and insulin adjustments in pediatric DKA. The simplicity and safety of this protocol make it a good option for managing this condition. Our study provides valuable insights; however, prospective multicenter trials are needed to confirm and broaden these findings.

## Supplementary Material

fmae003_Supplementary_Data
