# Supplementary material for: Revised one-bag IV fluid protocol for pediatric DKA: a feasible approach and retrospective comparative study
Source: J Trop Pediatr. 2024 Feb 9;70(2):fmae003. doi: 10.1093/tropej/fmae003 (PMC10858344; doi:10.1093/tropej/fmae003)
Supplement: fmae003_Supplementary_Data [file fmae003_supplementary_data.docx]

# Table. Characteristics of the sub-cohort groups; age, gender, and initial laboratory findings.

| **Characteristic** | **Sub-cohort: 2014 Traditional Protocol**  **(n = 61)** | **Sub-cohort: 2016 Revised Protocol**  **(n = 51)** | **p** |
| --- | --- | --- | --- |
| Age (years) | 12.33 (9.79-13.79) | 11.58 (6.9-13.4575) | 0.193 |
| Gender (M/F) | 22/29 | 31/29 | 0.374 |
| Glucose (mg/dL) | 470.0 (376.5-529.5) | 484.0 (407.5-567.0) | 0.632 |
| pH | 7.17 (7.09-7.21) | 7.17 (7.09-7.24) | 0.822 |
| Bicarbonate (mmol/L) | 9.7 (7.15-11.5) | 7.35 (4.85-10.95) | 0.027 |
| pCO2 | 23.86±6.37 | 21.92±8.07 | 0.150 |
| Na^+^ (mmol/L) | 131.0 (128.5-133.0) | 131.0 (128.0-133.25) | 0.502 |
| K^+^ (mmol/L) | 4.51 (4.11-5.07) | 4.6 (4.07-5.025) | 0.653 |
| Creatinine (mg/dL) | 1.0 (0.835-1.15) | 0.98 (0.84-1.19) | 0.827 |
|  |  |  |  |
| DKA severity (n, %) |  |  |  |
| Mild DKA | 17 (27.8) | 12 (23.5) |  |
| Moderate DKA | 18 (29.5) | 23 (45.1) | 0.170 |
| Severity DKA | 26 (42.6) | 16 (31.3) |  |

^*^The groups are designated sub-cohorts, each representing a one-year subset of a larger cohort study, to reflect the annual application of the protocols. Data were presented as median (25^th^-75^th^) or mean ± SD or n (%). A p-value < 0.05 is significant.
